# Frailty in rheumatoid arthritis and the general population: a cross-sectional analysis of the Groningen Frailty Indicator and Fried criteria

**DOI:** 10.1093/rheumatology/keag310

**Published:** 2026-06-17

**Authors:** Saskia P M Truijen, Annelies Boonen, Sofia Ramiro, Marloes van Onna

**Affiliations:** Department of Rheumatology, Maastricht University Medical Centre, Maastricht, The Netherlands; Care and Public Health Research Institute (CAPHRI), Maastricht University, Maastricht, The Netherlands; Department of Rheumatology, Maastricht University Medical Centre, Maastricht, The Netherlands; Care and Public Health Research Institute (CAPHRI), Maastricht University, Maastricht, The Netherlands; Department of Rheumatology, Leiden University Medical Centre, Leiden, The Netherlands; Department of Rheumatology, Zuyderland Medical Centre, Heerlen, The Netherlands; Department of Rheumatology, Maastricht University Medical Centre, Maastricht, The Netherlands; Care and Public Health Research Institute (CAPHRI), Maastricht University, Maastricht, The Netherlands

**Keywords:** frailty, ageing, rheumatoid arthritis, factors, cross-sectional analysis

## Abstract

**Objective:**

To compare frailty prevalence and factors driving frailty between older adults with RA and controls, using two conceptually distinct frailty instruments.

**Methods:**

Cross-sectional data from the Studying Ageing in Rheumatoid Arthritis (STAR) study were used, including 207 patients with RA and 214 population controls aged 55–85 years. Frailty was assessed using the Groningen Frailty Indicator (GFI; 15 yes/no items; physical, cognitive, social, psychological domains; frail ≥ 4/15) in all participants and the Fried criteria (five yes/no items; physical domain; prefrail = 1–2, frail ≥ 3, and combined frail/prefrail ≥ 1 deficit) in a clinically assessed subgroup (RA: *n* = 88, controls: *n* = 96). Prevalence and overlap between instruments were described by group. Uni- and multivariable logistic (GFI: frailty *vs* robust) and Poisson (Fried: frail/prefrail *vs* robust) regressions assessed effects of age, group, the age*group interaction and additional covariates.

**Results:**

Frailty prevalence was higher in RA than controls for GFI-frailty (34% *vs* 18%) and Fried-frailty/prefrailty (72% *vs* 50%). Among GFI-frail persons, 88% of patients with RA compared with 69% of controls were also frail or prefrail by Fried. Age was not associated with frailty in uni- or multivariable analyses for either instrument, in RA or controls (*p*_interaction age*group _>0.10). The association with RA became insignificant after multivariable adjustment. Living alone, comorbidity score ≥1, higher fatigue and anxiety were associated with GFI-frailty, and higher BMI and poorer physical function with Fried-frailty/prefrailty.

**Conclusion:**

Older adults with RA show higher (pre)frailty prevalence than controls, which is associated with disease-related consequences and vulnerability rather than age, questioning the added value of frailty assessment beyond routine clinical evaluation.

Rheumatology key messages

- Frailty in RA is largely consistent with disease manifestations and generic vulnerability, rather than age.
- The Groningen Frailty Indicator reflects broader biopsychosocial vulnerability, while the Fried criteria capture predominantly physical vulnerability.
- The added value of frailty assessment in RA beyond standard clinical evaluation is questionable.

## Introduction

The global population is ageing rapidly, with the proportion of individuals aged ≥65 years in the European Union expected to increase from one in five to one in three by 2050 [1]. The number of people aged ≥80 years is expected to triple between 2020 and 2050 [2]. Consequently, the proportion of individuals with chronic diseases, including RA, will rise [2–4]. Chronic systemic inflammation and age-related ‘inflammageing’ contribute to geriatric syndromes such as frailty [5, 6], with RA increasing the risk of developing frailty [7].

Frailty is characterized by decline in functioning across multiple physiological systems, resulting in reduced resilience and increased vulnerability to stressors [8, 9]. It is associated with adverse health outcomes including falls, disability, hospitalization and mortality [10–13]. Risk factors include demographic and social characteristics (e.g. advanced age, loneliness), clinical factors (e.g. multimorbidity) and lifestyle factors (e.g. physical inactivity) [14, 15].

Frailty assessment in RA remains challenging. The literature on frailty reveals highly variable prevalence estimates in RA (1.2% to 75.1%) [16–18], reflecting heterogeneity in studied populations and operationalization of frailty. Moreover, key features of frailty—such as fatigue, weakness and reduced physical function—overlap with RA manifestations, complicating the distinction between RA-related impairments and the physiological impact of ageing. This distinction is important as RA-related impairments require rheumatologic treatment, whereas geriatric frailty may require a broader geriatric perspective.

A commonly used frailty instrument is the examiner-assessed Fried frailty criteria [8], which conceptualizes frailty as a physical syndrome based on five components, including unintentional weight loss and reduced handgrip strength. Other instruments, such as the patient-reported Groningen Frailty Indicator (GFI) [19], adopt a multidimensional approach, incorporating physical, cognitive, psychological, and social domains. The Fried criteria—distinguishing frailty and prefrailty—have demonstrated both concurrent and predictive validity [20–22], showing agreement with other frailty measures and the ability to predict adverse outcomes such as fractures, hospitalization and mortality [21]. The GFI has also shown internal consistency and concurrent validity, with correlations to factors such as dependency and lower quality of life [23, 24], and a modest predictive value of mortality [25].

Few studies have included different frailty instruments within RA cohorts or examined whether (age-related) frailty patterns in RA differ from controls. Population controls allow understanding of whether age-related decline in RA is accelerated or similar to that of the general population, whether the same domains contribute to frailty in RA and controls, and to what extent frailty reflects disease-related *vs* age-related processes.

The Studying Ageing in Rheumatoid Arthritis (STAR) study was designed to examine ageing in patients with RA using population controls as a comparator. It includes the above-mentioned and conceptually different measures of frailty: the GFI and Fried criteria. Using cross-sectional data from STAR, the current study aimed to assess for each instrument [1] the prevalence of frailty and prefrailty and the contribution of frailty subdomains in RA and controls, and [2] factors driving (pre)frailty (i.e. prefrail or frail), with particular focus on the roles of age and RA.

## Methods

### Study design and population

A detailed description of the STAR cohort and measurements has been described previously [26, 27]. The STAR study is a cross-sectional observational study including patients with RA and population-based controls aged 55–85 years. Patients diagnosed with RA by their rheumatologist were recruited from the outpatient clinics of two large hospitals in the south of the Netherlands: the Maastricht University Medical Centre+ and the Zuyderland Medical Centre. Population controls were recruited through patients’ and employees’ friends or family members, snowball sampling and flyers. Presence of an inflammatory RMD or fibromyalgia was an exclusion criterion for population controls. Additional exclusion criteria for all participants were [1] severe comorbidity raising health concerns according to the treating rheumatologist or research team [2], a diagnosis of dementia or severe cognitive decline [3], life expectancy <2 years or [4] inability to understand the Dutch language or the study information.

**Figure 1:**
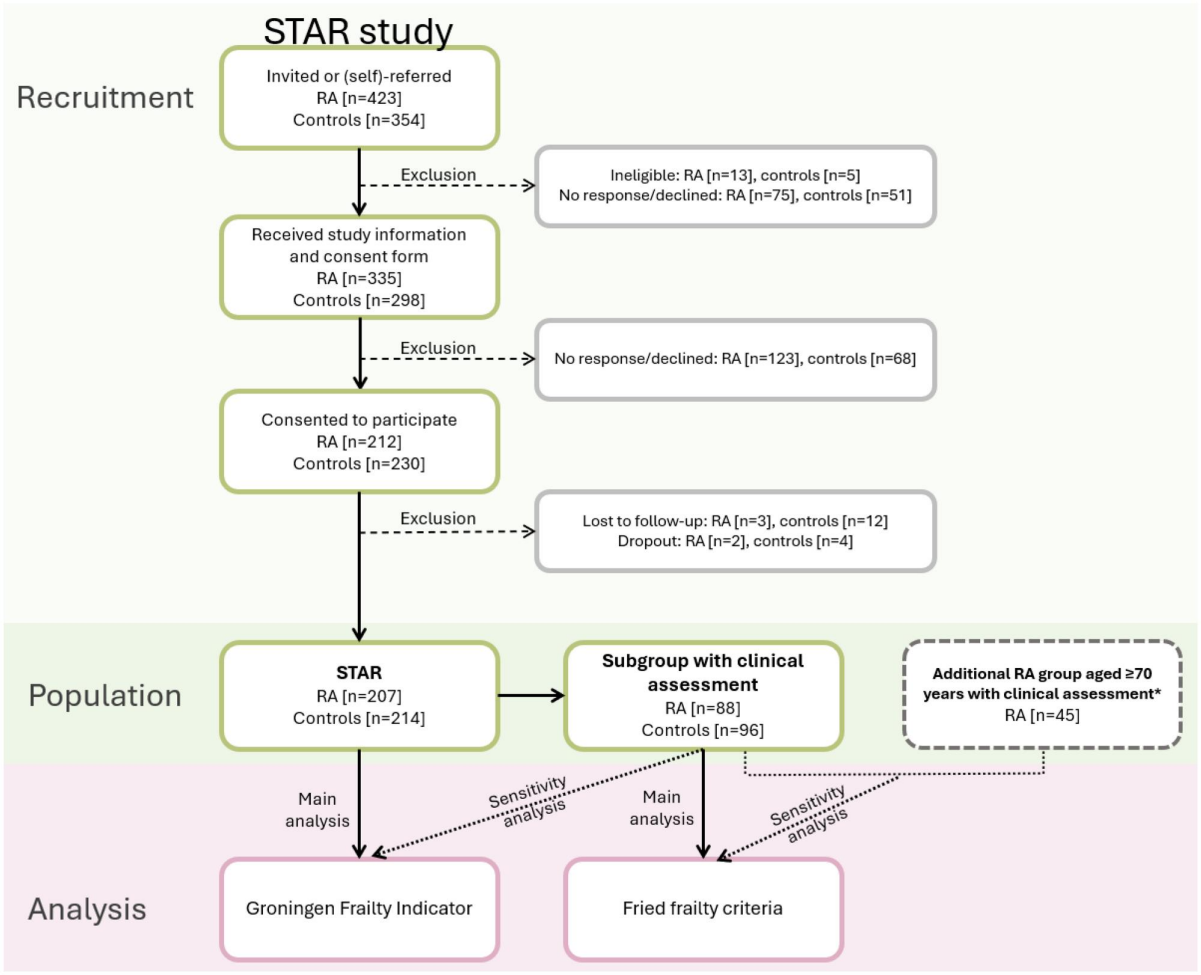
Flowchart of the STAR study. *Available outcome measure included only the Fried frailty criteria. The following covariates were not assessed in the additional RA group aged ≥70 years: marital status, MFI, RADAI, HAQ-DI, PHQ-9 and GAD-7. Abbreviations: STAR: Studying Ageing in Rheumatoid Arthritis; RA: rheumatoid arthritis; MFI: Multidimensional Fatigue Inventory; RADAI: Rheumatoid Arthritis Disease Activity Index; HAQ-DI: Health Assessment Questionnaire Disability Index; PHQ: Patient Health Questionnaire; GAD: generalized anxiety disorder

All participants underwent a telephone interview on medical history and medication use, followed by self-administered questionnaires at home. A subgroup of participants who were willing to attend the research centre underwent a clinical examination, performance tests and laboratory assessments. Within this subgroup, the target was 10 women and 10 men per five-year age interval (55–59, 60–64, 65–69, 70–74, 75–79, 80–85 years) to ensure adequate age and sex representation. However, recruitment for the full clinical examination of patients with RA aged ≥70 years proved challenging. Therefore, additional patients were enrolled under a reduced protocol in which basic characteristics and Fried criteria were assessed (additional RA group aged ≥70 years). These data were part of sensitivity analyses (Fig. 1). Assessments in this additional RA group ≥70 years took place immediately following their regular clinical visit, requiring no additional visit to the research centre.

The study was approved by the institutional medical ethical committee (NL71972.068.019) and all participants provided written informed consent. Recruitment took place between December 2022 and April 2025.

### Assessment of frailty

#### Groningen Frailty Indicator

The self-reported GFI was assessed in the total study population and consists of 15 items asking about problems related to four domains: physical (nine items on mobility, presence of comorbidity, fatigue, vision, hearing); cognitive (one item on problems with memory); social (two items on emotional isolation); and psychological (three items on depressed mood and feelings of anxiety) (Table S1) [19, 28]. Response categories were yes/no to indicate a problem or dependency, yielding a total score from 0 to 15. Individuals were classified as robust (score < 4) or frail (score ≥4) [19].

#### Fried frailty criteria

The Fried criteria were assessed by an examiner in the subgroup that underwent clinical assessment and consist of five criteria: unintentional weight loss; exhaustion; low physical activity; slow gait speed (adjusted for gender and height); and low handgrip strength (adjusted for gender and body mass index (BMI)) (Table S1) [8]. Individuals scoring positive (yes *vs* no) on one or two criteria were classified as prefrail and those with three or more positive criteria as frail. All others were considered robust. For analyses, frail and prefrail individuals were combined into one group (called frail/prefrail).

### Covariables

All participants completed self-administered questionnaires. Sociodemographic and lifestyle data included age (years), sex, highest achieved educational level [low (no education, primary education, or lower vocational education), middle (intermediate vocational education or higher secondary education), or high (higher vocational education or university education)], marital status [alone (single, divorced, or widower) or together (living together or married)], smoking status (never, former, current) and BMI. General health was rated on a visual analogue scale (VAS; 0 = very bad, 10 = very good), as was pain during the past week (0 = no pain, 10 = unbearable pain). Patients Global Assessment (PGA) of disease activity was recorded (VAS; 0 = very little, 10 = a lot) and adapted for controls by asking to score global joint complaints. Disability was assessed with the 20-item HAQ-Disability Index [HAQ-DI; 0 = no difficulty, 3 = unable to do; total score 0 (no disability) to 3 (maximum disability)] [29]. Fatigue was assessed with the 20-item Multidimensional Fatigue Inventory [MFI; 1 = yes, that is true, 5 = no, that is not true; total score 20 (no fatigue) to 100 (maximum fatigue)] [30]. The self-reported painful joint score was assessed by the Rheumatoid Arthritis Disease Activity Index [RADAI; 8 joint groups per side, 0 = no pain to 3 = severe pain per group; total score 0 (no joint pain) to 48 (severe pain in all joint groups)] [31]. The 9-item Patient Health Questionnaire (PHQ-9) assessed depressive symptoms [0 = not at all, 3 = nearly every day; total score 0 (no depression) to 27 (severe depression)] [32]. The 7-item General Anxiety Disorder scale (GAD-7) assessed anxiety symptoms [0 = never, 3 = nearly every day; total score 0 (no anxiety) to 21 (severe anxiety)] [33]. Because some participants aged 80–85 completed a shortened questionnaire package without the PHQ-9 and GAD-7, both measures were analysed as categorical variables with an additional ‘unknown’ category (0–4 = no or minimal; ≥5 = mild, moderate or severe; unknown).

Detailed information on current medication use and medical history was collected through a structured telephone interview and, when possible, cross-checked with participants’ electronic medical records (EMR). For patients with RA, current and past use of DMARDs, glucocorticoids and NSAIDs was recorded. The presence of erosive disease (yes/no) was assessed from the most recent hand and foot radiographs or, if unavailable, from the EMR. Comorbidities were classified using the Charlson Comorbidity Index (CCI; 0 = no comorbidities, 33 = maximum predicted mortality) [34]. For analysis, CCI scores were categorized as 0 or ≥1, excluding the presence of RA.

### Statistical analysis

The proportions of individuals classified as frail by the GFI and frail or prefrail by the Fried criteria were calculated for RA and controls with additional stratification by three age groups. In the subgroup with clinical assessment, overlap between GFI-based frailty and Fried-based frailty or prefrailty classifications was visualized using Venn diagrams among participants classified as frail or prefrail by either instrument. The Upsetplot package was used to illustrate the contribution and co-occurrence of frailty subdomains contributing to frailty, by displaying the proportion of frail or prefrail patients and controls meeting individual domains or combinations thereof [35]. Domains or combinations present in only one participant were omitted.

Manual forward multivariable logistic (GFI-frail) and Poisson regressions (Fried-frail/prefrail) assessed a potential differential effect of age on frailty between patients with RA and controls, and explored the role of additional covariates. As the prevalence of combined frailty/prefrailty by Fried was ≥50%, Poisson regression with robust standard errors was used to estimate prevalence ratios (PRs), as odds ratios (ORs) may overestimate associations when the event is frequent.

First, interaction terms age*group (RA/control) and age*sex were tested in models containing main effects. Models were stratified by group or sex if *p*_interaction _<0.10 and if the interaction was considered clinically relevant. The following covariables were further considered for the full multivariable models: educational level, marital status, smoking status, BMI, comorbidities (CCI), fatigue (MFI), self-reported no. and severity of painful joints (RADAI), self-reported pain levels (VAS pain), physical function (HAQ-DI), glucocorticoid use, depression (PHQ-9) and anxiety (GAD-7). Multicollinearity was assessed using variance inflation factors (VIF), with values above 10 indicating high multicollinearity and thus not included in the same model. Covariates were retained in the final model when they were significantly associated with the outcome (*P < *0.05) or were confounders in the relationship between age or group and the outcome (changing the coefficient >10% upon inclusion).

A first sensitivity analysis repeated the multivariable logistic regression for the GFI, only in the subgroup with clinical assessment, to gain insight into potential bias and the generalizability of findings (Fig. 1). To assess the impact of including a more representative sample of older RA patients, the additional group of patients aged ≥70 years was added to the clinically assessed subgroup. Fried-frailty and prefrailty prevalence was recalculated and uni- and multivariable regressions were repeated with available variables.

Statistical analyses were performed using STATA (StataCorp version 17).

## Results

### Study population

A total of 421 participants were included: 207 patients with RA [mean age 68 (SD 7) yrs; 62% women] and 214 population controls [mean age 68 (7) years; 60% women] (Fig. 1, Table 1). The subgroup who also underwent the clinical assessment consisted of 184 participants: 88 (*n* = 88/184; 48%) patients with RA [mean age 67 (8) years; 49% women] and 96 (*n* = 96/184; 52%) controls [mean age 68 (8) years; 55% women]. Compared with controls, patients with RA had a lower educational level (57% *vs* 41% finished low or middle education), were more often smokers (15% *vs* 6%) and had more often comorbidities (CCI score ≥1: 43% *vs* 30%). Controls within the subgroup that was also clinically assessed had worse physical health than controls who only completed questionnaires [mean SF36 PCS score 46 (11) *vs* 49 [8]] (Table S2).

**Table 1:** Characteristics of the total STAR study population and subgroup with clinical assessment.

	Total study population (n = 421)		Subgroup with clinical assessment (n = 184)	
	RA *n* = 207	Controls *n* = 214	RA *n* = 88	Controls *n* = 96
Age (yrs), mean (SD)	67.8 (7.3)	68.1 (7.1)	67.3 (7.8)	68.0 (7.7)
Sex, *n* women (%)	128 (62)	128 (60)	43 (49)	53 (55)
Educational level, *n* (%)^a^				
Low	46 (22)	20 (9)	18 (21)	11 (12)
Middle	73 (35)	69 (32)	35 (40)	28 (29)
High	86 (42)	123 (58)	34 (39)	56 (58)
Smoking status, *n* (%)				
Never	77 (37)	102 (48)	31 (35)	49 (51)
Former	100 (48)	100 (47)	47 (53)	41 (43)
Current	30 (15)	12 (6)	9 (10)	5 (5)
BMI (kg/m^2^), mean (SD)	26.5 (4.3)	26.5 (5.9)	27.0 (4.7)	27.7 (7.5)
Disease duration (yrs), median (IQR)	7.4 (3.1–16.4)	—	9.5 (3.3–19.8)	—
Erosive disease, *n* yes (%)^b^	57 (28)	—	35 (40)	—
Current RA treatment, *n* (%)				
csDMARDs	156 (75)	—	68 (77)	—
bDMARDs	74 (36)	—	34 (39)	—
tsDMARDs	4 (2)	—	1 (1)	—
Glucocorticoids	51 (25)	—	19 (22)	—
NSAIDs	43 (21)	—	22 (25)	—
RADAI score (0–48), median (IQR)^c^	10 (5–18)	3 (1–8)	11 (5–16)	4 (1–10)
General health (0–10), mean (SD)	6.5 (1.2)	7.4 (1.2)	6.4 (1.2)	7.3 (1.3)
PGA (0–10), mean (SD)	4.3 (2.4)	3.0 (2.7)	4.0 (2.2)	3.5 (2.9)
Self-rated pain last week (0–10), mean (SD)	4.1 (2.4)	2.7 (2.6)	4.0 (2.3)	3.2 (2.8)
HAQ-DI (0–3), median (IQR)^c^	0.6 (0.3–1.3)	0.1 (0–0.5)	0.6 (0.1–1.3)	0.1 (0–0.6)
MFI (20–100),^c^ median (IQR)	52 (41–63)	37 (28–54)	52 (44–63)	37 (29–56)
CCI (0–32), n *score* ≥1 (%)^d^	90 (43)	64 (30)	34 (39)	24 (25)
SF36 PCS score, mean (SD)	40.3 (9.7)	47.7 (9.5)	39.7 (9.4)	45.7 (10.5)
SF36 MCS score, mean (SD)	51.8 (9.6)	54.2 (7.4)	53.2 (9.0)	54.4 (6.8)

a Unknown educational level: *n* = 2 (RA) and *n* = 2 (controls) in total study population, *n* = 1 (RA) and *n* = 1 (controls) in subgroup;

b Unknown presence of erosive disease: *n* = 29 in total study population and *n* = 4 in subgroup;

c Unknown HAQ-DI, MFI and RADAI: *n* = 1 (RA) and *n* = 1 (controls);

d Rheumatic disease was not included in the CCI.

RA: rheumatoid arthritis; BMI: body mass index; csDMARDs: conventional synthetic disease-modifying anti-rheumatic drugs; bDMARDs: biologic disease-modifying anti-rheumatic drugs; tsDMARDs: targeted synthetic disease-modifying anti-rheumatic drugs; NSAID: non-steroidal anti-inflammatory drugs; RADAI: Rheumatoid Arthritis Disease Activity Index; PGA: Patient Global Assessment; HAQ-DI: Health Assessment Questionnaire Disability Index; MFI: Multidimensional Fatigue Inventory; CCI: Charlson Comorbidity Index; SF36 PCS: Short Form-36 Physical Component Summary; SF36 MCS: Short Form-36 Mental Component Summary.

### Prevalence of frailty

A higher proportion of patients with RA than controls was classified as frail by the GFI [RA: 34% (*n* = 70/207); controls: 18% (*n* = 38/214); *P < *0.01] as well as frail or prefrail by the Fried criteria [frail: RA: 7% (*n* = 6/88) *vs* controls: 5% (*n* = 5/96); prefrail: RA: 65% (*n* = 57/88); controls: 45% (*n* = 43/96); *P < *0.01] (Table 2). Across age categories, frailty was descriptively highest in those aged 75–85 years for both the GFI and Fried criteria, although this was less pronounced for GFI-frailty in controls (Table S3).

**Table 2:** Prevalence of frailty and prefrailty by the Groningen Frailty Indicator (GFI) and the Fried frailty criteria, including subdomains met among those classified as frail or prefrail.

	RA	Controls	

Groningen Frailty Indicator (*n* = 421)^a^	*n* = 207	*n* = 214	*P-*value^c^
GFI score (0–15), median (IQR)	2 (1–4)	1 (0–3)	<0.01*
Frailty, *n* (%)			<0.01*
Robust	137 (66)	176 (82)	
Frail	70 (34)	38 (18)	

**GFI subdomains (in the frail population)**	***n* = 70**	***n* = 38**	

Physical deficits, *n* ≥ 1 yes (%)	64 (91)	32 (84)	0.25
Mobility, *n* ≥ 1 yes (%)	16 (23)	4 (11)	0.12
Physical fitness, *n* score 0–6 (%)	48 (69)	18 (47)	0.03*
Vision problems, *n* yes (%)	18 (26)	4 (11)	0.06
Hearing problems, *n* yes (%)	21 (30)	17 (45)	0.13
Unintentional weight loss, *n* yes (%)	7 (10)	5 (14)	0.62
Polypharmacy, *n* yes (%)	56 (80)	15 (39)	<0.01*
Cognitive problems, *n* yes (%)	12 (17)	3 (8)	0.18
Social isolation, *n* ≥ 1 yes (%)	60 (86)	36 (95)	0.15
Psychological problems, *n* ≥ 1 yes (%)	61 (87)	30 (79)	0.26

	**RA**	**Controls**	

**Fried frailty criteria (*n* = 184)^b^**	***n* = 88**	***n* = 96**	***P -value* ^c^**

Fried criteria (0–5), median (IQR)	1 (0–2)	0.5 (0–1)	<0.01*
Frailty, *n* (%)			0.01*
Robust	25 (28)	48 (50)	
Prefrail	57 (65)	43 (45)	
Frail	6 (7)	5 (5)	

**Fried subdomains (in the frail/prefrail population)**	***n* = 63**	***n* = 48**	

Unintentional weight loss, *n* yes (%)	13 (21)	3 (6)	0.03*
Exhaustion, *n* yes (%)	38 (60)	35 (73)	0.16
Low level of physical activity, *n* yes (%)	5 (8)	7 (15)	0.26
Slow gait speed, *n* yes (%)	7 (11)	5 (10)	0.91
Low handgrip strength, *n* yes (%)	35 (56)	23 (48)	0.42

a In the total STAR population;

b In the subgroup with clinical assessment;

c Group differences (RA/control) were examined using *t* tests or Mann–Whitney *U* tests for continuous variables and χ^2^ tests for categorical variables;

* Statistically significant (*P-*value < 0.05).

RA: rheumatoid arthritis; GFI: Groningen Frailty Indicator.

**Figure 2:**
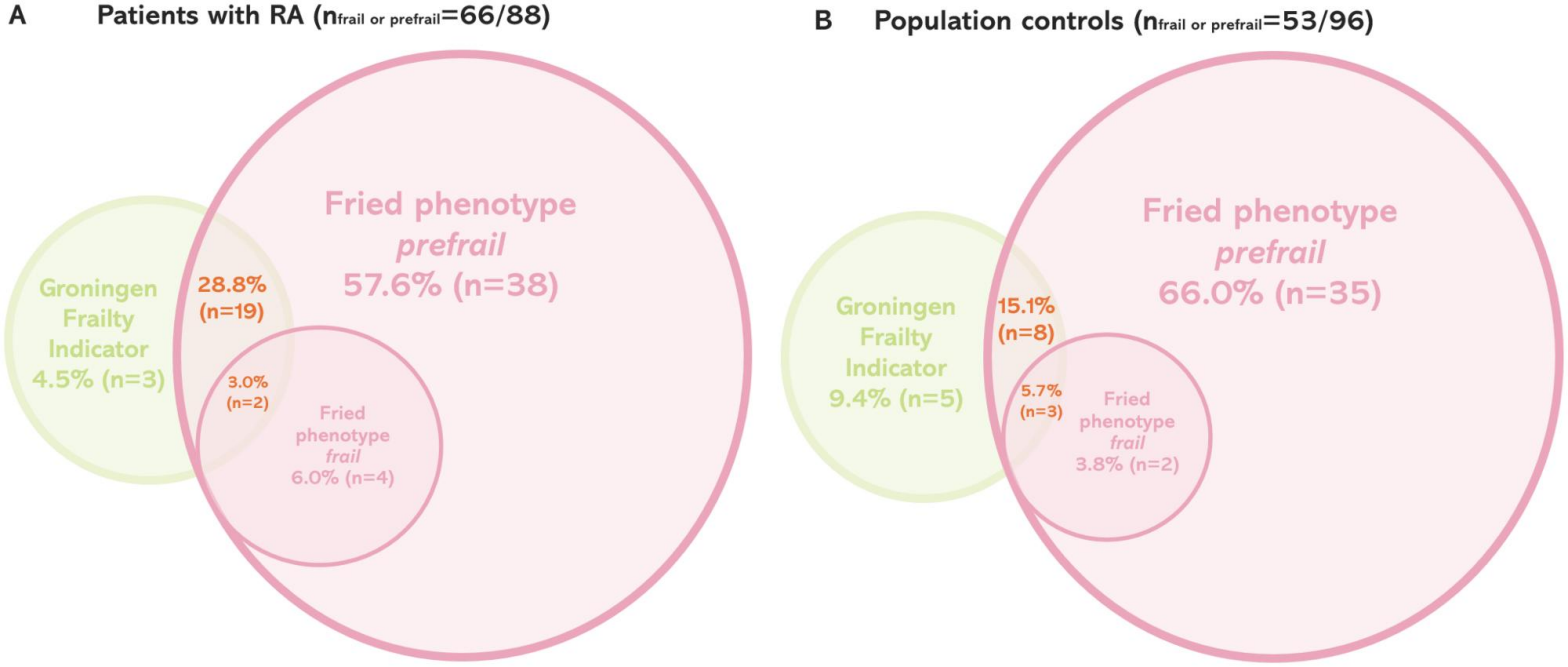
Overlap between GFI and Fried classifications among (**A**) patients with RA (*n* = 66) and (**B**) population controls (*n* = 53) classified as frail or prefrail in the subgroup with clinical assessment

Within the clinically assessed subgroup, GFI-frailty was lower in patients with RA than in the total RA population [27% (*n* = 24/88) *vs* 34%], but comparable among controls [16% (*n* = 16/96) *vs* 18%]. Overlap in prevalence between instruments was higher in patients with RA than in controls (Fig. 2A and B). Among patients with RA classified as frail by the GFI, 88% (*n* = 21/24) were also classified as frail or prefrail by the Fried criteria, predominantly due to prefrailty. In contrast, only 36% (*n* = 21/57) of those classified as frail or prefrail by the Fried criteria were also frail by the GFI. Corresponding proportions for controls were 69% (*n* = 11/16) and 23% (*n* = 11/48), respectively. Overall, patients with RA had 1.9 times (95%CI: 0.9–3.8) higher odds than controls of being classified as frail by the GFI compared with 2.5 times as frail/prefrail by the Fried (95%CI: 1.4–4.6).

### Domains of frailty

Among those classified as GFI-frail, frailty predominantly resulted from the accumulation of difficulties in the physical (91% *vs* 84%), social (86% *vs* 95%) and psychological (87% *vs* 79%) domains in patients and controls, respectively (Table 2 and Supplementary Fig. S1A and B). Within the physical domain, worse physical fitness (RA: 69%; controls: 47%) and polypharmacy (RA: 80%; controls: 39%) were the most contributing deficits and were more frequently reported by patients than controls. Among controls, hearing problems were also common (RA: 30%; controls: 45%). Cognitive problems were less often reported in both groups (RA: 17%; controls: 8%). Across age categories, psychological problems were less often reported by the oldest participants (Table S3).

Among those classified as Fried frail/prefrail, exhaustion (RA: 60%; controls: 73%) and low handgrip strength (RA: 56%; controls: 48%) were the most contributing deficits in both groups (Table 2), either alone or in combination (Supplementary Fig. S1C and D). Patients with RA reported unintentional weight loss more often than controls (RA: 21%; controls: 6%).

### Factors associated with frailty

Age was not significantly associated with frailty/prefrailty in uni- nor multivariable analyses for either instrument, and the effect of age did not differ between patients with RA and controls or between men and women (*p*_interactions_>0.10) (Table 3 and Table S4). RA was associated with GFI-frailty (OR = 2.4, 95%CI: 1.5–3.8) and Fried-frailty/prefrailty (PR = 1.4, 95%CI: 1.1–1.8) in univariable analyses, but these associations were no longer observed after adjustment for confounders. Factors associated with GFI-frailty included living alone (OR = 2.6, 95%CI: 1.3–5.1), a comorbidity score of ≥1 (OR = 2.0, 95%CI: 1.1–3.6), a higher MFI score (OR = 1.04, 95%CI: 1.02–1.07) and mild to severe anxiety (OR = 4.1, 95%CI: 2.0–8.5) (Table 3). Factors associated with Fried-frailty/prefrailty were higher BMI (PR = 1.02, 95%CI: 1.01–1.03) and HAQ-DI (PR = 1.4, 95%CI: 1.1–1.7).

**Table 3:** Associations between age, group (RA *vs* controls) and GFI-frailty or Fried-frailty/prefrailty, adjusted for sociodemographic and clinical factors: multivariable logistic and Poisson regression.

	**Groningen frailty indicator Frail (≥4) vs. robust (<4) (*n* = 421)** ^a^		Fried criteria Prefrail or frail (≥1) vs. robust 0 (*n* = 184)^b^	
	OR	(95% CI)	PR	(95% CI)
Group, patient with RA	0.97	0.52–1.82	1.13	0.91–1.40
Age, years^c^	1.03	0.98–1.07	0.99	0.98–1.01
Sex, women	1.85	0.97–3.51	0.86	0.68–1.09
Marital status, alone vs together	**2.61** ^e^	**1.34–5.07**	—	
BMI, kg/m^2^	—		**1.02^e^**	**1.01–1.03**
CCI (0–32), ≥1 vs 0	**1.95** ^d^ ^,e^	**1.05–3.62**	1.08^e^	0.85–1.37
MFI [20–100]	**1.04** ^d^ ^,e^	**1.02–1.07**	1.006^d^^,e^	0.998–1.014
RADAI score (0–48)	1.03^d^^,e^	0.99–1.06	1.01^e^	1.00–1.02
HAQ-DI (0–3)	—		**1.40^e^**	**1.13–1.73**
Glucocorticoids use, yes	1.82^d^	0.89–3.73	—	
PHQ-9^f^, mild, moderate, or severe vs no-minimal depression	1.68^e^	0.79–3.56	—	
GAD-7^f^, mild, moderate, or severe vs no-minimal anxiety	**4.07** ^e^	**1.95–8.49**	—	

a Educational level, smoking status, BMI, VAS pain and HAQ-DI were no significant predictors or confounders of age and/or group for the GFI;

b educational level, marital status, smoking status, VAS pain, glucocorticoid use, PHQ-9 and GAD-7 were no significant predictors or confounders of age and/or group for the Fried criteria;

c there were no confounding variables in the association between age and the outcome measure (ΔOR_age_ ≥ 10%);

d confounding variable in the association between group and the outcome measure (ΔOR_group_ ≥ 10%);

e associated with the outcome measure (*P < *0.05);

f unknown category not presented. **Statistically significant (*P*-value < 0.05)**.

OR: odds ratio; PR: prevalence ratio; RA: rheumatoid arthritis; BMI: body mass index; CCI: Charlson Comorbidity Index; MFI: Multidimensional Fatigue Inventory; RADAI: Rheumatoid Arthritis Disease Activity Index; VAS: Visual Analogue Scale; HAQ-DI: Health Assessment Questionnaire Disability Index; PHQ: Patient Health Questionnaire; GAD: generalized anxiety disorder.

### Sensitivity analyses

Within the clinically assessed subgroup, RA was not associated with GFI-frailty in univariable analysis (OR = 1.9, 95%CI: 0.9–3.8). In multivariable analysis, a comorbidity score of ≥1 was no longer associated with GFI-frailty, and the effect size of marital status became stronger (OR = 4.4, 95%CI: 1.4–13.2) (Table S5).

The additional RA group aged ≥70 years included 41 patients [mean age 76 (9) years; 59% women]. They had more often a comorbidity than the main RA population (CCI score ≥1: 54% *vs* 43%) (Table S2). Including this group in the analyses of the Fried criteria did not substantially affect the proportion classified as prefrail [from 65% (*n* = 57/88)% to 61% (*n* = 78/129)], but increased the proportion classified as frail from 7% (*n* = 6/88)% to 16% (*n* = 21/129). Specifically, more patients had a low level of physical activity, slow gait speed and low handgrip strength (Table S6). In contrast to the main analyses, age was associated with Fried-frailty/prefrailty in univariable analyses (PR = 1.01, 95%CI: 1.00–1.02); however, this association was no longer observed after adjustment for group and sex (Table S7). Owing to the absence of data on key covariates (MFI, RADAI and HAQ-DI), no further analyses could be performed.

## Discussion

Frailty and prefrailty were more common in older adults with RA than in population controls according to both the GFI and the Fried criteria. The Fried criteria—focusing on the physical frailty phenotype—classified a larger proportion of patients with RA than controls as frail/prefrail compared with the GFI, although actual Fried-frailty was rare (RA: 7%; controls: 5%). Notably, age was not associated with frailty or prefrailty in either group. After adjustment for RA-related consequences, such as multimorbidity, fatigue or pain, RA was no longer independently associated with frailty/prefrailty. This suggests that higher frailty prevalence in RA may largely reflect the overlap between frailty determinants and disease-related consequences of RA, rather than representing a distinct geriatric syndrome.

Previous studies consistently report higher frailty prevalence in RA than in the general population, with estimates strongly dependent on the instrument used [7, 16, 18, 36–39]. Instruments incorporating psychological and social domains yield higher prevalence than those focusing solely on the physical domain [16, 36]. Direct comparisons of frailty instruments between patients with RA and population controls remain scarce [7, 40]. Within RA populations, several studies have evaluated multiple frailty instruments [7, 37, 41, 42], but none have directly compared the GFI with the Fried phenotype. In a UK Biobank study using modified Fried criteria in a younger population (mean age 59 years), frailty prevalence was 3.4% in controls and 19% in RA [7], higher than in our clinically assessed subgroup (7%), possibly reflecting selection of relatively healthier RA patients, as including patients aged ≥70 years increased Fried-defined frailty to 16%.

Although frailty is typically considered a geriatric syndrome increasing with advancing age [6, 36, 40], age was not associated with frailty for either instrument in our main analyses, neither in patients with RA nor in controls. This aligns with a Japanese study in older patients with RA [43], the UK Biobank study of patients with RA aged 40–69 years [7] and a previous systematic literature review [16], all reporting no clear increase in frailty with age. Importantly, the lack of association in our study was also evident in controls. The absence of an age-by-RA interaction further suggests no accelerated age-related frailty development in RA within the studied age range. When including patients aged ≥70 years, a univariable association with age emerged but disappeared after adjustment for RA.

The higher prevalence of frailty and prefrailty in RA compared with controls according to both the GFI and Fried criteria in crude analyses was largely associated by typical manifestations of RA including fatigue, joint pain and related functional limitations. Although more prevalent and severe in RA, these factors were also often present in controls, supporting that frailty reflects an accumulation of deficits. As expected, psychosocial factors (e.g. anxiety, living alone) were associated with the multidimensional GFI, while physical factors (HAQ-DI, BMI) were associated with the physically oriented Fried criteria. These factors were largely consistent with previous studies in RA and general older populations [15, 43–46].

Notably, 88% of patients classified as frail by the GFI were also frail/prefrail according to the Fried criteria. While firm conclusions cannot be drawn, this may suggest that GFI-frailty reflects more a prefrailty state rather than actual frailty. Overlap may be explained by high correspondence between the item ‘general fitness’ in the GFI and the ‘exhaustion’ subdomain of the Fried criteria. It is possible that Fried frailty underestimates true frailty. This also suggests that Fried prefrailty may overestimated, as meeting a single criterion—such as exhaustion—could reflect consequences of RA rather than the frailty syndrome itself.

Several limitations should be acknowledged. First, relatively healthier patients with RA may have been more likely to participate, potentially introducing selection bias. Sensitivity analyses including additional older patients (≥70 years) who declined full participation increased the prevalence of Fried-frailty from 7% to 16%, suggesting underestimation of frailty in the main analyses and indicating that frailty may be more pronounced at more advanced ages. Conversely, selection bias among controls in the clinically assessed subgroup may have favoured less physically healthy individuals, potentially attenuating differences between patients with RA and controls, which may be an explanation for the absence of a univariable association between RA and GFI-defined frailty in sensitivity analyses. The recruitment of population controls through convenience methods, including snowball sampling and flyers, may also limit the generalizability of findings to the broader general population. Second, the cross-sectional design limits conclusions regarding age-related frailty progression and precludes causal inference. Third, the absence of key covariates in the additional RA group aged ≥70 years precluded full multivariable modelling in the sensitivity analyses for the Fried criteria in the group enriched by persons with RA ≥70 years. As HAQ-DI was a significant predictor of Fried-frailty/prefrailty in the main analyses, the sensitivity analyses are likely to underestimate the contribution of disease-related factors. Moreover, as the additional RA group included only patients and no controls, the interaction between age and group could not be examined in this sensitivity analysis. In conclusion, the GFI and Fried criteria provide different frailty estimates in older patients with RA. While the GFI primarily reflects broader biopsychosocial vulnerability, the Fried criteria capture predominantly physical vulnerability. The higher frailty prevalence in RA compared with controls is consistent with increased disease-related physical consequences and psychosocial vulnerabilities that also affect the general older population. The prefrailty category within the Fried criteria may overestimate vulnerability in RA, as patients frequently meet one or two physical criteria in the context of disease-related consequences. The absence of an association between age and frailty or prefrailty and overlap with consequences of RA raises questions about the added value of frailty assessment beyond routine disease evaluation in clinical practice. Further longitudinal analyses are needed to understand the performance of different frailty instruments. As the Fried criteria incorporates two subdomains that point to sarcopenia (grip strength and gait speed), the Fried Criteria might better predict other geriatric outcomes such as falls, loss of independence or mortality in longitudinal analyses. Meanwhile, optimizing disease control remains critical in older patients with RA, with additional attention to geriatric principles (e.g. sarcopenia) when challenges arise beyond disease control.

## Supplementary Material

keag310_Supplementary_Data

## Data Availability

The data underlying this article will be shared on reasonable request to the corresponding author.
