# Supplementary material for: Frailty in rheumatoid arthritis and the general population: a cross-sectional analysis of the Groningen Frailty Indicator and Fried criteria
Source: Rheumatology (Oxford). 2026 Jun 17;65(7):keag310. doi: 10.1093/rheumatology/keag310 (PMC13335654; doi:10.1093/rheumatology/keag310)
Supplement: keag310_Supplementary_Data [file keag310_supplementary_data.docx]

**Frailty in rheumatoid arthritis and the general population: A cross-sectional analysis of the Groningen Frailty Indicator and Fried criteria – Supplementary Material**

| Supplementary Table S1. Description of the Groningen Frailty Indicator (GFI) and Fried frailty criteria | | | |
| --- | --- | --- | --- |
| **Groningen Frailty Indicator (score 0–15)** ^a^ | | **Fried frailty criteria ^b^** | |
| Physical domain | *Mobility*: Independently (Q1) doing groceries, (Q2) walk outside the house, (Q3) dressing, (Q4) going to the toilet  *Physical health*: (Q5) Self-reported physical health score between 0-6 or 7-10  *Vision*: (Q6) Problems in daily life due to poor vision  *Hearing:* (Q7) Problems in daily life due to difficulty hearing  *Nutrition:* (Q8) Unintentional substantial weight loss in the past six months  *Morbidity:* (Q9) Use of ≥4 medications (polypharmacy) | Unintentional weight loss | “Yes” to question “Have you unintentionally lost ≥4.5 kg or ≥5% of body weight during previous year?” |
|  |  | Exhaustion | “Yes” to CES-D item “I felt that everything I did was an effort” or “I could not get going”.[1] |
|  |  | Low physical activity | “Yes” to question “Do you sit more than four hours per day, go on a walk less than once per month, and not engage in activities such as cycling or jogging?”[2] This definition was reported to correspond to an energy expenditure of <393 kcal/week for men and <280 kcal/week for women. |
|  |  | Slow gait speed | Gender- and height-specific thresholds for slow gait speed at 4-meter walk test (men: height ≤173 cm: ≥6.1 seconds; height >173 cm: ≥5.3 seconds, women: height ≤159 cm: ≥6.1 seconds; height >159: ≥5.3 seconds).[3] |
| Cognitive domain | (Q10) Memory complaints | Low handgrip strength | Gender- and BMI-specific thresholds for low HGS using a calibrated digital Jamar handheld dynameter.[4] Low HGS, defined as the lowest 20% of values of population-based older adults, was determined using gender- and BMI-specific thresholds (men: BMI ≤24: HGS ≤29 kg; BMI 24.1–26.0: HGS ≤30 kg; BMI 26.1–28.0: HGS ≤30 kg; BMI >28.0: HGS ≤32 kg, women: BMI ≤23: HGS ≤17 kg; BMI 23.1–26.0: HGS ≤17.3 kg; BMI 26.1–29.0 HGS ≤18 kg; BMI >29.0: HGS ≤21 kg).[3] |
| Social domain | (Q11) Experience emptiness, (Q12) missing people around, (Q13) feeling abandoned sometimes |  |  |
| Psychological domain | (Q14) Feeling down-hearted or sad lately, (Q15) Feeling nervous or anxious lately |  |  |
| **GFI** Groningen Frailty Indicator; **CES-D** Center for Epidemiologic Studies Depression Scale; **BMI** Body Mass Index; **HGS** Handgrip Strength  ^a^ Each of the 15 items is scored as no (0 points) or yes (1 point); ^b^ Each of the 5 criteria is scored as not met (0 points) or met (1 point). | | | |

| Supplementary Table S2. Comparison of characteristics between the total study population excluding the subgroup with clinical assessment to the subgroup with clinical assessment, and characteristics of the additional RA group aged **≥70 years for sensitivity analyses** | | | | | | | |
| --- | --- | --- | --- | --- | --- | --- | --- |
|  | **Total study population without subgroup with clinical assessment** | **Subgroup with clinical assessment** |  | **Additional RA group aged ≥70 years** | **Total study population without subgroup with clinical assessment** | **Subgroup with clinical assessment** |  |
|  | RA  n=120 | RA  n=87 | *p*-value ^a^ | RA n=41 | Control  n=119 | Control  n=95 | *p*-value ^a^ |
| Age (yrs), mean (SD) | 68.1 (6.9) | 67.3 (7.8) | 0.44 | 76.3 (9.2) | 68.2 (6.7) | 68.1 (7.6) | 0.94 |
| Sex, n *women* (%) | 85 (71) | 43 (49) | <0.01* | 24 (59) | 75 (63) | 53 (56) | 0.28 |
| Educational level, n (%) ^b^ |  |  | 0.41 |  |  |  | 0.72 |
| Low | 28 (23) | 18 (21) |  | 9 (22) | 9 (8) | 11 (12) |  |
| Middle | 38 (32) | 35 (40) |  | 19 (46) | 41 (34) | 28 (29) |  |
| High | 52 (43) | 34 (39) |  | 10 (24) | 68 (57) | 55 (58) |  |
| Smoking status, n (%) |  |  | 0.24 |  |  |  | 0.59 |
| Never | 46 (38) | 31 (36) |  | 12 (29) | 53 (45) | 49 (52) |  |
| Former | 53 (44) | 47 (54) |  | 26 (63) | 59 (50) | 41 (43) |  |
| Current | 21 (18) | 9 (10) |  | 3 (7) | 7 (6) | 5 (5) |  |
| BMI (kg/m^2^), mean (SD) | 26.0 (3.9) | 27.1 (4.7) | 0.09 | 26.5 (4) | 25.5 (4.0) | 27.7 (7.6) | <0.01* |
| Disease duration (yrs), median (IQR) | 7.3 (2.9–13.2) | 9.8 (3.3–19.8) | 0.11 | 18 (10–31) | - | - | - |
| Erosive disease, n *yes* (%) ^c^ | 23 (19) | 34 (39) | 0.02* | 22 (54) | - | - | - |
| Current RA treatment, n (%) |  |  |  |  |  |  |  |
| csDMARDs | 89 (74) | 67 (77) | 0.64 | 25 (61) | - | - | - |
| bDMARDs | 41 (34) | 34 (38) | 0.58 | 17 (42) | - | - | - |
| tsDMARDs | 3 (3) | 1 (1) | 0.49 | 1 (2) | - | - | - |
| Glucocorticoids | 32 (27) | 19 (22) | 0.43 | 14 (34) | - | - | - |
| NSAIDs | 21 (18) | 22 (25) | 0.17 | 7 (17) | - | - | - |
| RADAI score (0–48), median (IQR) | 10 (4–20) | 11 (5–16) | 0.90 | - | 3 (1–6) | 4 (1–10) | 0.35 |
| Self-rated general health (0–10), mean (SD) | 6.5 (1.3) | 6.4 (1.2) | 0.95 | 5.9 (2) | 7.5 (1.2) | 7.3 (1.3) | 0.28 |
| PGA (0–10), mean (SD) | 4.5 (2.5) | 4.0 (2.2) | 0.21 | 3.7 (3) | 2.6 (2.5) | 3.5 (2.9) | 0.01* |
| VAS pain (0–10), mean (SD) | 4.2 (2.4) | 4.0 (2.3) | 0.54 | 4.3 (3) | 2.3 (2.3) | 3.3 (2.8) | <0.01* |
| MFI (20–100), median (IQR) ^d^ | 54 (38–64) | 52 (44–63) | 0.70 | - | 35 (27–50) | 37 (29–56) | 0.38 |
| CCI (0–32), n *score* ≥1 (%) ^e^ | 56 (47) | 34 (39) | 0.28 | 22 (54) | 40 (34) | 24 (25) | 0.19 |
| HAQ-DI (0–3), median (IQR) | 0.6 (0.3–1.3) | 0.6 (0.1–1.3) | 0.79 | - | 0.1 (0–0.4) | 0.1 (0.0–0.6) | 0.28 |
| SF36 PCS score, mean (SD) | 40.7 (9.9) | 39.7 (9.4) | 0.48 | - | 49.4 (8.2) | 45.7 (10.5) | <0.01* |
| SF36 MCS score, mean (SD) | 50.7 (9.9) | 53.2 (9.0) | 0.06 | - | 54.1 (7.9) | 54.4 (6.8) | 0.73 |
| ^a^ Differences were examined using t-tests or Mann-Whitney U tests for continuous variables and chi-square tests for categorical variables; ^b^ Unknown educational level: n=2 (RA) and n=1 (controls) in total study population, n=1 (controls) in subgroup; ^c^ Unknown presence of erosive disease: n=25 in total study population, n=4 in subgroup, n=3 in additional RA group aged ≥70 years; ^d^ Unknown MFI: n=1 (RA) and n=13 (controls) in subgroup; ^e^ Rheumatic disease was not included in the CCI; *Statistically significant (*p*-value<0.05) **RA** Rheumatoid Arthritis; **BMI** Body Mass Index; **csDMARDs** Conventional Synthetic Disease-Modifying Anti-Rheumatic Drugs; **bDMARDs** Biologic Disease-Modifying Anti-Rheumatic Drugs; **tsDMARDs** Targeted Synthetic Disease-Modifying Anti-Rheumatic Drugs; **NSAID** Non-Steroidal Anti-Inflammatory Drugs; **RADAI** Rheumatoid Arthritis Disease Activity Index; **PGA** Patient Global Assessment; **MFI** Multidimensional Fatigue Inventory; **CCI** Charlson Comorbidity Index; **HAQ-DI** Health Assessment Questionnaire Disability Index;  **SF36 PCS** Short Form-36 Physical Component Summary; **SF36 MCS** Short Form-36 Mental Component Summary; **GFI** Groningen Frailty Indicator | | | | | | | |


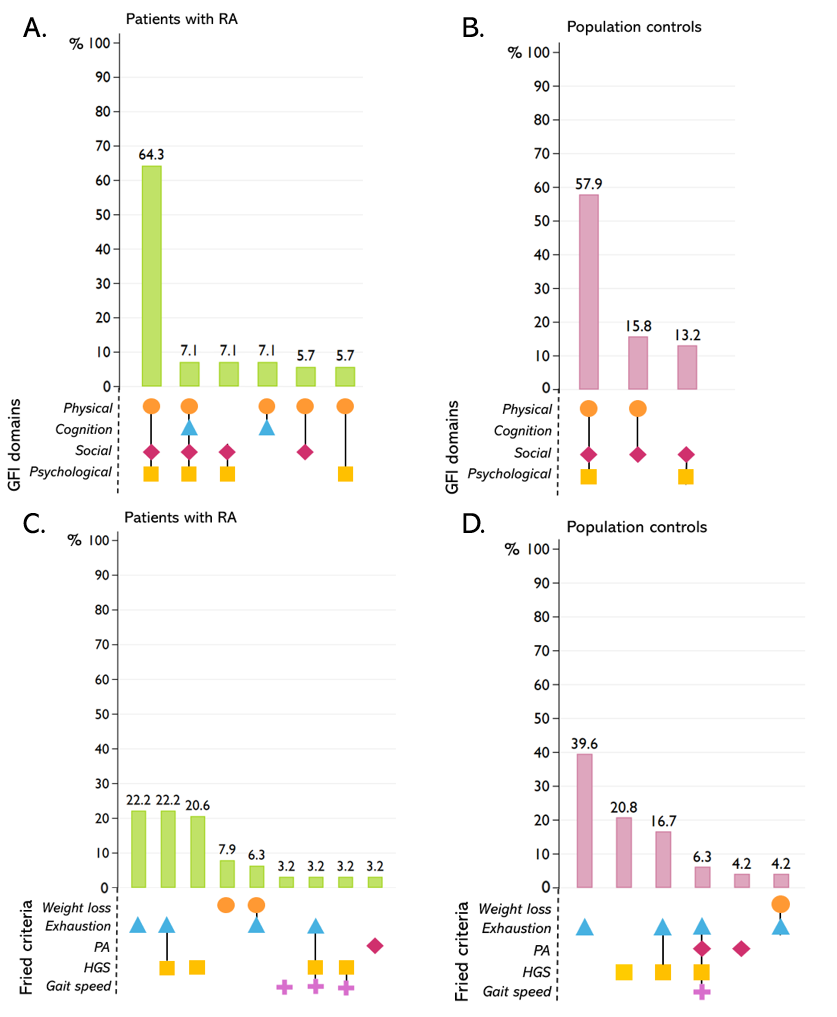


Supplementary Figure S1A-D. Proportion (%) of frail or prefrail patients with RA (GFI n=70; Fried n=63) and population controls (GFI n=38; Fried n=48) fulfilling individual GFI (A-B) or Fried (C-D) subdomains. Combinations occurring in only one participant were omitted.
Interpretation: For the GFI, combined deficits in the physical, social, and psychological domains were common contributors to frailty (64% of patients; 58% of controls). For the Fried criteria, exhaustion and HGS, alone or combined, were the predominant contributors to frailty/prefrailty (exhaustion only: 22% of patients with RA, exhaustion + low HGS: 22%, low HGS only: 21%; exhaustion only: 40% of controls, low HGS only: 21%, and exhaustion + low HGS: 17%).
Abbreviations: RA Rheumatoid Arthritis; GFI Groningen Frailty Indicator; PA Physical Activity; HGS Handgrip Strength

| Supplementary Table S3. Prevalence of frailty by the Groningen Frailty Indicator (GFI) and frailty or prefrailty by the Fried frailty criteria, including subdomains met among those classified as frail/prefrail | | | | | | | | |
| --- | --- | --- | --- | --- | --- | --- | --- | --- |
|  | **RA** | | | | **Controls** | | | |
| **Groningen Frailty Indicator (n=421) ^a^** | Total n=207 | 55-64 yrs n=70 | 65-74 yrs n=98 | 75-85 yrs n=39 | Total n=214 | 55-64 yrs n=67 | 65-74 yrs n=105 | 75-85 yrs n=42 |
| Robust, n (%) | 137 (66) | 47 (67) | 69 (70) | 21 (54) | 176 (82) | 56 (84) | 87 (83) | 33 (79) |
| Frail, n (%) | 70 (34) | 23 (33) | 29 (30) | 18 (46) | 38 (18) | 11 (16) | 18 (17) | 9 (21) |
|  |  |  |  |  |  |  |  |  |
| **GFI subdomains (in the frail population)** | n=70 | n=23 | n=29 | n=18 |  |  |  |  |
| Physical deficits, n *≥1 yes* (%) | 64 (90) | 18 (78) | 25 (86) | 18 (100) | 32 (84) | 9 (82) | 14 (78) | 9 (100) |
| Mobility, n *≥1 yes* (%) | 16 (23) | 3 (13) | 8 (28) | 5 (28) | 4 (11) | 0 (0) | 2 (11) | 2 (22) |
| Physical fitness, n *score 0-6* (%) | 48 (67) | 11 (48) | 20 (69) | 17 (94) | 18 (47) | 6 (55) | 5 (28) | 7 (78) |
| Vision problems, n *yes* (%) | 18 (25) | 7 (30) | 6 (21) | 5 (28) | 4 (11) | 2 (18) | 1 (6) | 1 (11) |
| Hearing problems, n *yes* (%) | 21 (30) | 5 (22) | 9 (31) | 7 (39) | 17 (45) | 4 (36) | 5 (28) | 8 (89) |
| Unintentional weight loss, n *yes* (%) | 7 (10) | 2 (9) | 2 (7) | 3 (17) | 5 (13) | 0 (0) | 4 (22) | 1 (11) |
| Polypharmacy, n *yes* (%) | 56 (79) | 15 (65) | 26 (90) | 15 (83) | 15 (39) | 3 (27) | 6 (33) | 6 (67) |
| Cognitive problems, n *yes* (%) | 12 (17) | 6 (26) | 3 (10) | 3 (17) | 3 (8) | 0 (0) | 2 (11) | 1 (11) |
| Social isolation, n *≥1 yes* (%) | 60 (85) | 19 (83) | 25 (86) | 16 (89) | 36 (95) | 10 (91) | 18 (100) | 8 (89) |
| Psychological problems, n *≥1 yes* (%) | 61 (86) | 22 (96) | 27 (93) | 12 (67) | 30 (79) | 11 (100) | 15 (83) | 4 (44) |
|  | **RA** | | | | **Controls** | | | |
| **Fried frailty criteria (n=184) ^b^** | Total n=88 | 55-64 yrs n=34 | 65-74 yrs n=36 | 75-85 yrs n=18 | Total n=96 | 55-64 yrs n=36 | 65-74 yrs n=37 | 75-85 yrs n=23 |
| Robust, n (%) | 25 (28) | 11 (33) | 7 (19) | 7 (39) | 48 (50) | 17 (47) | 23 (62) | 8 (35) |
| Prefrail, n (%) | 57 (65) | 20 (59) | 29 (81) | 8 (44) | 43 (45) | 19 (53) | 13 (35) | 11 (48) |
| Frail, n (%) | 6 (7) | 3 (9) | 0 (0) | 3 (17) | 5 (5) | 0 (0) | 1 (3) | 4 (17) |
|  |  |  |  |  |  |  |  |  |
| **Fried subdomains (in the frail/prefrail population)** |  |  |  |  |  |  |  |  |
| Unintentional weight loss, n *yes* (%) | 13 (21) | 7 (30) | 4 (14) | 2 (18) | 3 (6) | 2 (11) | 0 (0) | 1 (7) |
| Exhaustion, n *yes* (%) | 38 (60) | 17 (74) | 14 (48) | 7 (64) | 35 (73) | 17 (89) | 7 (50) | 11 (73) |
| Low level of physical activity, n *yes* (%) | 5 (8) | 2 (9) | 2 (7) | 1 (9) | 7 (15) | 1 (5) | 2 (14) | 4 (27) |
| Slow gait speed, n *yes* (%) | 7 (11) | 1 (4) | 3 (10) | 3 (27) | 5 (10) | 0 (0) | 1 (7) | 4 (27) |
| Low handgrip strength, n *yes* (%) | 35 (56) | 8 (35) | 16 (55) | 11 (100) | 23 (48) | 4 (21) | 9 (64) | 10 (67) |
| **^a^** In the total STAR population; **^b^** In the subgroup with clinical assessment; **GFI** Groningen Frailty Indicator; **RA** Rheumatoid Arthritis | | | | | | | | |

| Supplementary Table S4. Associations between sociodemographic and clinical factors and GFI-frailty or Fried-frailty/prefrailty – univariable logistic and Poisson regression | | | | |
| --- | --- | --- | --- | --- |
|  | **Groningen Frailty Indicator Frail (≥4) vs. robust (<4)**  **(n=421)** | | **Fried criteria   Prefrail or frail (≥1) vs. robust (0) (n=184)** | |
|  | OR | (95% CI) | PR | (95% CI) |
| Group, *patient with RA* | **2.4** | 1.5–3.8 | **1.4** | 1.1–1.8 |
| Age, *years* | 1.02 | 0.99–1.05 | 1.00 | 0.98–1.02 |
| Sex, *women* | **1.7** | 1.1–2.7 | 1.0 | 0.8–1.3 |
| Educational level |  |  |  |  |
| Low | *reference* | | *reference* | |
| Middle | 1.3 | 0.7–2.5 | 0.9 | 0.7–1.1 |
| High | 0.8 | 0.5–1.6 | **0.7** | 0.5–1.0 |
| Marital status, *alone vs. together* | **3.1** | 1.9–5.1 | 1.2 | 0.9–1.5 |
| Smoking status |  |  |  |  |
| No | *reference* | | *reference* | |
| Former | 0.8 | 0.5–1.2 | 1.1 | 0.9–1.4 |
| Current | 1.7 | 0.8–3.4 | 1.1 | 0.7–1.7 |
| BMI, *kg/m^2^* | 1.01 | 0.97–1.05 | **1.02** | 1.01–1.03 |
| CCI (0–32), *≥1 vs. 0* | **3.0** | 1.9–4.7 | **1.4** | 1.1–1.7 |
| MFI (20–100) | **1.08** | 1.06–1.10 | **1.02** | 1.01–1.03 |
| RADAI score (0–48) | **1.10** | 1.07–1.12 | **1.03** | 1.02–1.05 |
| VAS pain (0–10) | **1.3** | 1.2–1.5 | **1.12** | 1.07–1.17 |
| HAQ-DI (0–3) | **4.3** | 3.0–6.3 | **1.7** | 1.5–1.9 |
| Glucocorticoids use, *yes* | **3.3** | 2.0–5.5 | **1.5** | 1.3–1.9 |
| PHQ-9 ^a,c^, *mild, moderate, or severe vs. no-minimal depression* | **10.6** | 6.4–17.7 | **1.5** | 1.2–1.9 |
| GAD-7 ^b,c^, *mild, moderate, or severe vs. no-minimal anxiety* | **10.4** | 6.0–18.0 | 1.1 | 0.8–1.5 |
| ^a^ Unknown PHQ-9: n=11 (GFI) and n=8 (Fried); ^b^ Unknown GAD-7: n=17 (GFI) and n=10 (Fried); ^c^ Unknown category not presented. **Statistically significant (*p*-value<0.05)**  **OR** Odds Ratio; **PR** Prevalence Ratio; **RA** Rheumatoid Arthritis; **BMI** Body Mass Index; **CCI** Charlson Comorbidity Index; **MFI** Multidimensional Fatigue Inventory; **RADAI** Rheumatoid Arthritis Disease Activity Index; **VAS** Visual Analogue Scale; **HAQ-DI** Health Assessment Questionnaire Disability Index; **PHQ** Patient Health Questionnaire; **GAD** Generalized Anxiety Disorder | | | | |

| Supplementary Table S5. Sensitivity analysis: Associations between age, group (RA vs. controls), and GFI-frailty in the subgroup with clinical assessment, adjusted for sociodemographic and clinical factors – multivariable logistic regression | | |
| --- | --- | --- |
|  | **Groningen Frailty Indicator   Frail (≥4) vs. robust (<4) (n=184) ^a,b^** | |
|  | OR | (95% CI) |
| Group, *patient with RA* | 1.24 | 0.51–2.97 |
| Age, *years* | 1.01 | 0.95–1.08 |
| Sex, *women* | 1.54 | 0.63–3.79 |
| Marital status, *alone vs. together* | **4.36 ^d^** | 1.44–13.2 |
| CCI (0–32), *≥1 vs. 0* | 2.11 ^c,d^ | 0.82–5.44 |
| MFI (20–100) | **1.05 ^c,d^** | 1.01–1.08 |
| RADAI score (0–48) | 1.03 ^c,d^ | 0.99–1.06 |
| GAD-7 ^e^, *mild, moderate, or severe vs. no-minimal anxiety* | **4.63 ^d^** | 1.68–12.8 |
| ^a^ Educational level, smoking status, BMI, RADAI, VAS pain, HAQ-DI, glucocorticoid use, and the PHQ-9 were no significant predictors or confounders of age and/or group for the GFI; ^b^ There were no confounding variables in the association between age and the outcome measure (∆OR_age_≥10%); ^c^ Confounding variable in the association between group and the outcome measure (∆OR_group_≥10%); ^d^ Associated with the outcome measure (*p*<0.05); ^e^ Unknown category not presented. **Statistically significant (*p*-value<0.05)**  **OR** Odds Ratio; **RA** Rheumatoid Arthritis; **BMI** Body Mass Index; **CCI** Charlson Comorbidity Index; **MFI** Multidimensional Fatigue Inventory; **RADAI** Rheumatoid Arthritis Disease Activity Index; **VAS** Visual Analogue Scale; **HAQ-DI** Health Assessment Questionnaire Disability Index; **PHQ** Patient Health Questionnaire; **GAD** Generalized Anxiety Disorder | | |

| Supplementary Table S6. Sensitivity analysis: Prevalence of frailty or prefrailty and subdomains by the Fried frailty criteria in the subgroup with clinical assessment including the additional RA group aged ≥70 years | | | |
| --- | --- | --- | --- |
|  | **RA** | **Controls** |  |
| **Fried frailty criteria (n=225)** | n=129 | n=96 | *p-*value ^a^ |
| Fried criteria (0–5), median (IQR) | 1 (0–2) | 0.5 (0–1) | <0.01* |
| Frailty, n (%) |  |  | <0.01* |
| Robust | 30 (24) | 48 (50) |  |
| Prefrail | 78 (61) | 43 (45) |  |
| Frail | 21 (16) | 5 (5) |  |
|  |  |  |  |
| **Subdomains** |  |  |  |
| Unintentional weight loss, n *yes* (%) | 20 (20) | 3 (6) | 0.03* |
| Exhaustion, n *yes* (%) | 60 (61) | 35 (73) | 0.14 |
| Low level of physical activity, n *yes* (%) ^2^ | 15 (15) | 7 (15) | 0.78 |
| Slow gait speed, n *yes* (%) | 18 (18) | 5 (10) | 0.36 |
| Low handgrip strength, n *yes* (%) ^2^ | 64 (65) | 23 (48) | 0.05 |
| ^a^ Group differences (RA/control) were examined using chi-square tests; ^b^ Unknown low level of physical activity and low handgrip strength: n=1 (RA); *Statistically significant (*p*-value<0.05)  **RA** Rheumatoid Arthritis | | | |

| Supplementary Table S7. Associations between sociodemographic and clinical factors and Fried-frailty/prefrailty in the subgroup with clinical assessment including the additional RA group aged ≥70 years – uni- and multivariable Poisson regression | | | | |
| --- | --- | --- | --- | --- |
|  | **Fried criteria   Prefrail or frail (≥1) vs. robust (0) (n=225)** | | **Fried criteria   Prefrail or frail (≥1) vs. robust (0) (n=225)** | |
|  | Univariable | | Multivariable ^1^ | |
|  | PR | (95% CI) | PR | (95% CI) |
| Group, *patient with RA* | **1.5** | 1.3–1.9 | **1.5** | 1.2–1.9 |
| Age, *years* | **1.01** | 1.00–1.02 | 1.009 | 0.999–1.019 |
| Sex, *women* | 1.0 | 0.8–1.2 | 1.03 | 0.86–1.24 |
| Educational level |  |  | - | |
| Low | *reference* | |  | |
| Middle | 0.9 | 0.7–1.1 |  | |
| High | **0.7** | 0.6–0.9 |  | |
| Marital status, *alone vs. together* | ꝉ | | ꝉ | |
| Smoking status |  |  | - | |
| No | *reference* | |  | |
| Former | 1.1 | 0.9–1.4 |  | |
| Current | 1.2 | 0.8–1.6 |  | |
| BMI, *kg/m^2^* | **1.02** | 1.01–1.03 | - | |
| CCI (0–32), *≥1 vs. 0* | **1.4** | 1.1–1.6 | - | |
| MFI (20–100) | ꝉ | | ꝉ | |
| RADAI score (0–48) | ꝉ | | ꝉ | |
| VAS pain (0–10) | **1.09** | 1.05–1.14 | - | |
| HAQ-DI (0–3) | ꝉ | | ꝉ | |
| Glucocorticoids use, *yes* | **1.5** | 1.2–1.7 | - | |
| PHQ-9, *mild, moderate, or severe vs. no-minimal depression* | ꝉ | | ꝉ | |
| GAD-7, *mild, moderate, or severe vs. no-minimal anxiety* | ꝉ | | ꝉ | |
| ^1^ The multivariable model only included group, age, and sex as key covariates were not measured in the additional RA group aged ≥70 years (referred to as ꝉ). **Statistically significant (*p*-value<0.05) RA** Rheumatoid Arthritis; **PR** Prevalence Ratio; **BMI** Body Mass Index; **CCI** Charlson Comorbidity Index; **MFI** Multidimensional Fatigue Inventory; **RADAI** Rheumatoid Arthritis Disease Activity Index; **VAS** Visual Analogue Scale; **HAQ-DI** Health Assessment Questionnaire Disability Index; **PHQ** Patient Health Questionnaire; **GAD** Generalized Anxiety Disorder | | | | |

**References supplementary material**

[1] Radloff LS. The CES-D scale: A self-report depression scale for research in the general population. Applied psychological measurement 1977;1(3):385-401

[2] ‘Frailty' bij ouderen (NTVG); 2009. Available from: <https://www.ntvg.nl/artikelen/frailty-bij-ouderen>. [Accessed November 24th 2025]

[3] Fried LP, Tangen CM, Walston J, Newman AB, Hirsch C, Gottdiener J, et al. Frailty in older adults: evidence for a phenotype. J Gerontol A Biol Sci Med Sci 2001;56(3):M146-56.

[4] Truijen SPM, Boonen A, Ramiro S, van Onna M. Physical function in older people with RA and population controls: A cross-sectional study of self-reported ability and objective performance measures (under review). 2025.
